# Effect of *Astragalus membranaceus* on left ventricular remodeling in HFrEF: a systematic review and meta-analysis

**DOI:** 10.3389/fphar.2024.1345797

**Published:** 2024-01-12

**Authors:** Xu Han, Ting Yu, Xi Chen, Zhiyan Du, Man Yu, Jiang Xiong

**Affiliations:** ^1^ Chongqing Changshou Traditional Chinese Medicine Hospital, Chongqing, China; ^2^ Traditional Chinese Medicine Hospital Dianjiang Chongqing, Chongqing, China

**Keywords:** *Astragalus membranaceus*, heart failure with reduced ejection fraction, left ventricular remodeling, randomized controlled trials, systematic review, meta-analysis

## Abstract

**Background:** Left ventricular remodeling (LVR) is a key factor leading to the onset and progression of heart failure with reduced ejection fraction (HFrEF). Improving LVR can delay the progression of HFrEF and improve quality of life.

**Objective:** To evaluate the improvement effect of *Astragalus membranaceus (A. membranaceus)* on LVR in patients with HFrEF.

**Method:** We retrieved randomized controlled trials (RCTs) of *A. membranaceus* in treating HFrEF from eight Chinese and English databases, up until 31 October 2023. To assess the quality of the literature, we utilized the bias risk tool from the Cochrane Handbook. For meta-analysis, we employed Review Manager 5.4.1 software. Additionally, we performed sensitivity analysis and publication bias assessment using Stata 17.0 software.

**Result:** Totally 1,565 patients were included in 19 RCTs. Compared to conventional treatment (CT), the combination therapy of *A. membranaceus* with CT demonstrated significant improvements in LVR, specifically increasing left ventricular ejection fraction (LVEF, MD = 5.82, 95% CI: 4.61 to 7.03, *p* < 0.00001), decreasing left ventricular end-diastolic diameter (LVEDD, MD = −4.05, 95% CI: −6.09 to −2.01, *p* = 0.0001), and left ventricular end-systolic diameter (LVESD, MD = −12.24, 95% CI: −15.24 to −9.24, *p* < 0.00001). The combination therapy of *A. membranaceus* with CT also improved clinical efficacy (RR = 4.81, 95% CI: 3.31 to 7.00, *p* < 0.00001), reduced brain natriuretic peptide (BNP, MD = −113.57, 95% CI: −146.91 to −81.22, *p* < 0.00001) level, and increased 6-min walking distance (6-MWD, MD = 67.62, 95% CI: 41.63 to 93.60, *p* < 0.00001). In addition, the combination therapy of *A. membranaceus* with CT mitigated inflammatory responses by reducing tumor necrosis factor-alpha (TNF-α, MD = −16.83, 95% CI: −22.96 to −10.71, *p* < 0.00001), interleukin-6 (IL-6, MD = −29.19, 95% CI: −36.08 to −22.30, *p* < 0.00001), and high-sensitivity C-reactive protein (hs-CRP, MD = −0.98, 95% CI: −1.43 to −0.52, *p* < 0.0001). Notably, the combination therapy of *A. membranaceus* with CT did not increase the incidence of adverse reactions (RR = 0.86, 95% CI: 0.25 to 2.96, *p* = 0.81).

**Conclusion:** This systematic review and meta-analysis revealed that the combination therapy of *A. membranaceus* with CT has more advantages than CT alone in improving LVR and clinical efficacy in HFrEF patients, without increasing the incidence of adverse reactions. However, due to the limited quality of included studies, more high-quality investigations are required to provide reliable evidence for clinical use.

**Systematic Review Registration:** https://www.crd.york.ac.uk/PROSPERO/display_record.php?RecordID=397571, Identifier: CRD42023397571.

## 1 Introduction

Heart failure (HF) is a major public health concern worldwide, with a high incidence and mortality rate (Roger, 2021). Reduced left ventricular ejection fraction (LVEF) is responsible for approximately 50% of HF cases, which are classified as heart failure with reduced ejection fraction (HFrEF) (Lavalle et al., 2023). HFrEF is typically characterized by a baseline LVEF ≤40% and pathological ventricular dilation, known as left ventricular remodeling (LVR) (Bloom et al., 2017). This remodeling process refers to the structural and functional changes in the left ventricle following a myocardial infarction or chronic pressure overload (Aimo et al., 2019). These changes include ventricular size, shape, and muscle structure alterations, leading to diastolic and systolic dysfunction. Over time, these transformations can result in progressive HF, significantly contributing to morbidity and mortality in HF patients (Stencel et al., 2023). Recent studies have shown that interventions targeting LVR can have a significant impact on clinical outcomes in HFrEF (Biering-Sørensen et al., 2020; Lee et al., 2021). Several drugs, such as angiotensin-converting enzyme inhibitor (ACEI), angiotensin II receptor blocker (ARB), and beta-blocker, have been demonstrated to slow down or even reverse LVR to some extent (Xie et al., 2023; Álvarez-Zaballos and Martínez-Sellés, 2023). However, their efficacy is often limited by side effects and the inability to fully restore ventricular function. As a result, there is a continuous need for novel therapeutic agents that can more effectively prevent and treat LVR in HFrEF.

*Astragalus membranaceus (A. membranaceus)*, a versatile Chinese herbal medicine with both medicinal and edible properties, is a popular therapeutic option for addressing cardiovascular diseases, including HF (Wang et al., 2023). *A. membranaceus* granules and *A. membranaceus* injection are derived from *A. membranaceus* and contain a range of effective active ingredients, including flavonoids, saponins, polysaccharides, as well as amino acids and trace elements (Zhu et al., 2022). Numerous basic and clinical research studies have uncovered the potential benefits of *A. membranaceus* in preventing and reversing LVR, as well as enhancing cardiac function and clinical outcomes in HFrEF patients (Dai et al., 2023; Ren et al., 2023). The potential mechanisms behind *A. membranaceus*’s actions include improving cardiac function, antioxidant properties, anti-inflammatory effects, anti-fibrotic properties, and reducing ventricular remodeling (Wang et al., 2023). Yang et al. (2013) found that Astragaloside IV, the main component of *A. membranaceus*, can inhibit the TLR4/NF -κ B signaling pathway, thereby reducing the inflammatory response and improving ISO-induced myocardial hypertrophy and fibrosis. Similarly, Ma et al. (2011) reported that *A. membranaceus* extract has the potential to increase blood lipid levels, inhibit lipid peroxidation, enhance antioxidant enzyme activity, and reduce the risk of hyperlipidemia and oxidative stress-related coronary heart disease. While most published studies have consistently demonstrated the efficacy of *A. membranaceus* when combined with conventional therapy for HFrEF, there is still a need for evidence-based medicine to validate its effectiveness in improving LVR. Thus, we performed a meta-analysis to quantify the overall impact of *A. membranaceus* on LVR and clinical efficacy.

## 2 Methods

### 2.1 Study registration

The PRISMA (Preferred Reporting Items for Systematic Reviews and Meta-analysis) guidelines were followed in conducting this study (Hutton et al., 2015). The study protocol was registered with PROSPERO (registration number: CRD42023397571), ensuring full disclosure and transparency in our research process.

### 2.2 Database and search strategy

This study systematically searched multiple databases, including PubMed, Embase, Cochrane Library, Web of Science, China Knowledge Infrastructure Database (CNKI), Wanfang Data, China Biomedical Database (CBM), and China Science and Technology Journal Database (VIP), from their establishment to 31 October 2023. To ensure comprehensive coverage, we also manually searched the reference lists of published literature. In terms of search strategies, we used “title/keywords” searches such as “*Astragalus membranaceus*”, “*radix astragali*”, “*huang qi*”, “heart failure”, and “left ventricular remodeling”. Our search included various forms of *A. membranaceus* preparations, including granules, injections, boiled soup, and extracts. To increase search accuracy, we employed Boolean logical operators (e.g., AND, OR) for strategic combinations. As people in China use Chinese characters to search for entries in electronic databases, we adapted our search methods accordingly. Throughout the search process, we adhered to the PRISMA guidelines to ensure the retrieval of comprehensive and accurate studies.

### 2.3 Inclusion criteria

The inclusion criteria for our study were carefully constructed using the PICOS approach, which considers study design, participants, interventions, comparators, and outcomes (Hutton et al., 2015). The specific inclusion criteria were as follows:

1) Study design: Randomized controlled trials (RCTs), published in English or Chinese, were selected, with a primary focus on original research.
2) Participants: Patients were diagnosed with HF, aged 18 years or older, and possessing a LVEF ≤40%.
3) Interventions: *A. membranaceus* administered orally or intravenously as a single treatment, or in combination with standard HF conventional treatment.
4) Comparators: Comparators were placebo or standard HF conventional treatment.
5) Outcomes: Primary outcomes concentrated on LVR, encompassing LVEF, LVEDD, and LVESD. Secondary outcomes encompassed clinical efficacy, BNP level, 6-MWD, and pro-inflammatory cytokines (TNF-α, IL-6, hs-CRP).

### 2.4 Exclusion criteria

The following criteria were implemented to exclude studies:

1) Study design: Non-randomized studies, case reports, reviews, cell or animal studies.
2) Participants: Patients with unstable HF, those aged below 18 years, or with a LVEF >40%.
3) Interventions: Studies involving *A. membranaceus* administration via routes other than oral or intravenous infusion.
4) Comparators: Studies using unconventional comparators, such as non-placebo control groups or unproven alternative therapies.
5) Outcomes: Studies with incomplete or inconsistent outcomes, such as those focusing on non-LVR parameters or including non-validated clinical efficacy assessments.
6) Duplicate publications: Studies that were previously published (only the most comprehensive data should be selected) or were currently under review elsewhere.
7) Unavailable full text: Studies whose full text could not be accessed online or via email.

### 2.5 Study selection and data extraction

All identified studies were managed using Endnote 20.5 software, after which duplicates were eliminated. Two reviewers (XH and TY) independently examined titles and abstracts to eliminate the irrelevant studies. Full-text versions of these studies were then downloaded and reviewed to identify potentially eligible studies. Once the eligible studies were identified, two reviewers (XH and TY) independently extracted data from each included study. The extracted information included details such as the first author, publication year, mean age, sex, sample size, interventions, treatment duration, and outcome indicators. Any disagreements during the data extraction process were resolved through discussion and consensus, ensuring accuracy and consistency.

### 2.6 Quality assessment

According to the Cochrane Handbook (Cumpston et al., 2019), two reviewers (XC and ZD) conducted an independent quality assessment for the included studies. They evaluated seven crucial items: random sequence generation, allocation concealment, participant and personnel blinding, outcome assessment blinding, incomplete outcome data, selective reporting, and other potential biases. Each item was meticulously categorized as low, unclear, or high risk of bias. The “risk of bias” summary and corresponding graph offered a comprehensive overview. Disagreements were resolved via the involvement of a third author (JX), ensuring a balanced and unbiased evaluation.

### 2.7 Data analysis

Revman version 5.4.1 software was employed for conducting meta-analysis. Relative risk (RR) for dichotomous variables and mean difference (MD) for continuous variables were selected as the measures of effect size, accompanied by a 95% confidence interval (CI) for interval estimation. When the heterogeneity between studies was minimal (*p* ≥ 0.1, *I* ^*2*^ ≤ 50%), a fixed effects model was chosen for analysis. If feasible, a subgroup analysis was conducted based on different administration methods or treatment duration to further explore the underlying factors that might influence the results. Sensitivity analysis and publication bias assessment were performed using Stata 17.0 software.

## 3 Results

### 3.1 Literature screening

**FIGURE 1:**
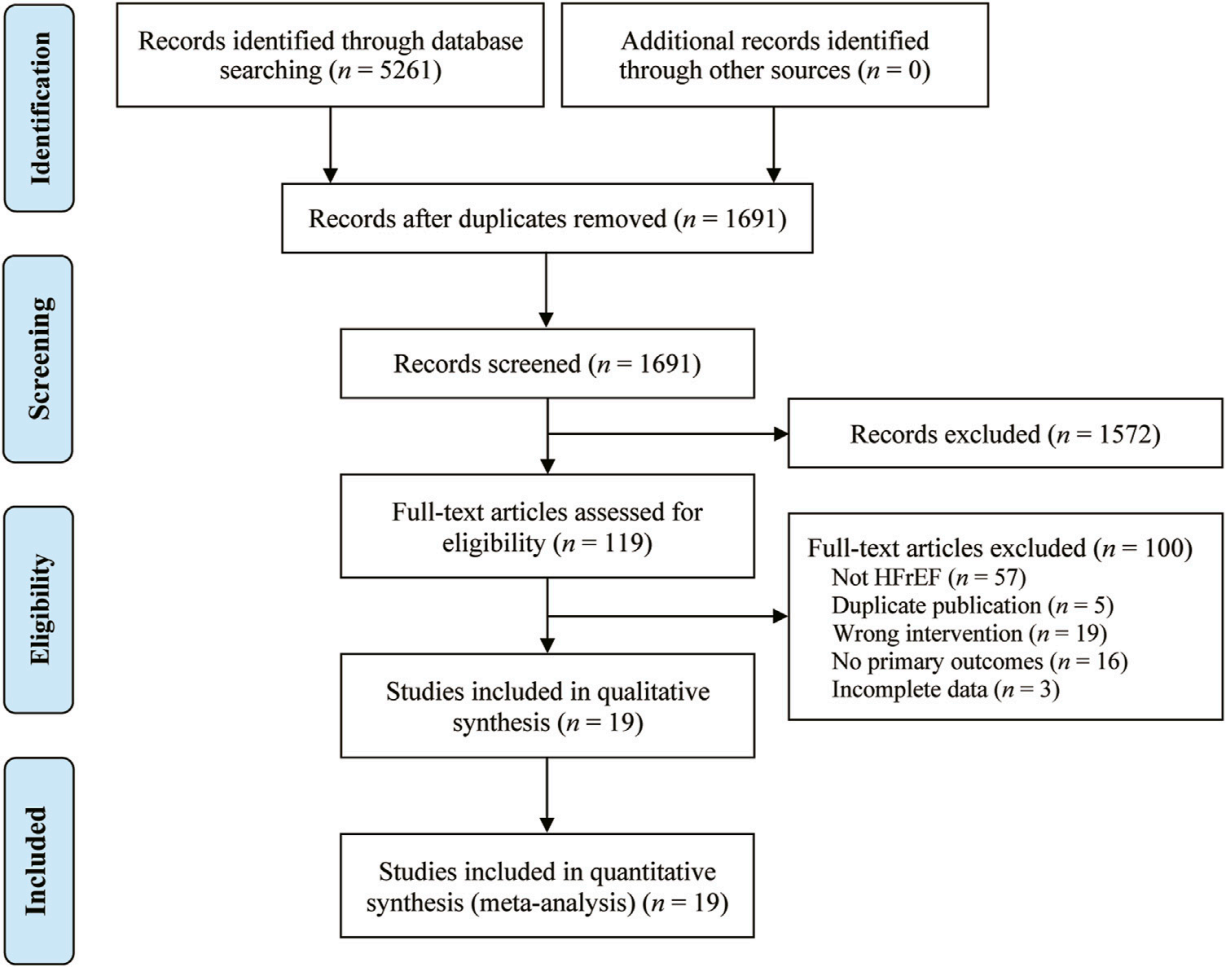
The PRISMA study flowchart of study search.

We conducted a systematic search of the database and retrieved 5,261 potentially relevant original studies, including PubMed (*n* = 62), Embase (*n* = 56), Cochrane library (*n* = 29), Web of Science (*n* = 48), CNKI (*n* = 1,499), Wanfang Data (*n* = 1,501), VIP (*n* = 644), and CBM (*n* = 1,422). Of these, 3,570 duplicate studies were excluded using Endnote 20.5 software. Following this, 1,572 studies were deleted after reviewing their titles and abstracts. After reading 119 studies in full, 100 were further excluded. Eventually, a total of 19 original studies (Wu and Chen, 2005; Zhang et al., 2005; Feng and He, 2008; Meng and Ma, 2008; Liu, 2010; Wang et al., 2010; Yang et al., 2010; Zhang and Li, 2014; Huang et al., 2016; Shen et al., 2016; Zhong et al., 2016; Zhu et al., 2016; Chen et al., 2018; Wei, 2018; Gan, 2019; Lv et al., 2019; Xian, 2019; Yang and Tu, 2019; Yuan et al., 2019) were included in the analysis. The study selection is visually represented in Figure 1.

### 3.2 Study characteristics

Table 1 presents the key characteristics of the 19 included studies. All these studies were conducted in China and published between 2005 and 2019. A total of 1,565 patients (856 males and 697 females) were enrolled, with sample sizes ranging from 23 to 78. The treatment period varied between 2 weeks and 1 month. The control group received conventional treatment (CT) for HFrEF as recommended in HF treatment guidelines, which included ACEI, ARB, β-receptor blockers, diuretics, spironolactones, and drugs that enhance myocardial metabolism. The treatment group received *A. membranaceus* in combination with CT. The following outcomes were reported across the included studies: LVEF (Wu and Chen, 2005; Zhang et al., 2005; Feng and He, 2008; Meng and Ma, 2008; Liu, 2010; Wang et al., 2010; Yang et al., 2010; Zhang and Li, 2014; Huang et al., 2016; Zhong et al., 2016; Zhu et al., 2016; Chen et al., 2018; Wei, 2018; Gan, 2019; Lv et al., 2019; Xian, 2019; Yang and Tu, 2019; Yuan et al., 2019), LVEDD (Huang et al., 2016; Zhu et al., 2016; Wei, 2018; Xian, 2019; Yang and Tu, 2019), LVESD (Huang et al., 2016; Zhu et al., 2016; Wei, 2018; Xian, 2019; Yang and Tu, 2019), clinical efficacy (Wu and Chen, 2005; Feng and He, 2008; Meng and Ma, 2008; Liu, 2010; Wang et al., 2010; Zhang and Li, 2014; Zhu et al., 2016; Chen et al., 2018; Wei, 2018; Gan, 2019; Lv et al., 2019; Xian, 2019; Yang and Tu, 2019; Yuan et al., 2019), BNP (Wang et al., 2010; Shen et al., 2016; Zhong et al., 2016; Zhu et al., 2016; Chen et al., 2018; Gan, 2019; Lv et al., 2019; Xian, 2019; Yang and Tu, 2019; Yuan et al., 2019), 6-MWD (Meng and Ma, 2008; Yang et al., 2010; Zhong et al., 2016; Chen et al., 2018; Wei, 2018; Lv et al., 2019; Yuan et al., 2019), TNF-α (Wu and Chen, 2005; Zhang et al., 2005; Yang et al., 2010; Zhang and Li, 2014; Huang et al., 2016; Shen et al., 2016; Xian, 2019), IL-6 (Zhang et al., 2005; Zhang and Li, 2014; Huang et al., 2016; Shen et al., 2016; Lv et al., 2019), and hs-CRP levels (Huang et al., 2016; Lv et al., 2019; Xian, 2019). Of the 19 studies, 7 studies (Wu and Chen, 2005; Meng and Ma, 2008; Wang et al., 2010; Yang et al., 2010; Chen et al., 2018; Wei, 2018; Xian, 2019) reported adverse events, with 4 studies (Wang et al., 2010; Chen et al., 2018; Xian, 2019) of them reporting no adverse events.

**TABLE 1:** Study characteristics.

First author (publication year)	Sample size		NYHA classification						Mean age (years)		Sex (M/F)		Interventions		*A. membranaceus* content	CT drugs	Treatment duration	Outcomes
	T	C	T			C			T	C	T	C	T	C				
Chen et al. (2018)	23	23	4	9	10	4	10	9	50.7 ± 6.7	51.5 ± 6.8	14/9	12/11	*A. membranaceus* granules, 30 g, bid + CT	CT	100 g	Diuretic, ACEI, carvedilol	3 W	①④⑤⑥⑩
Feng and He. (2008)	34	32	16	13	5	14	15	3	57.83 ± 7.79	57.93 ± 7.71	15/17	18/16	*A. membranaceus* injection, 40 mL, qd + CT	CT	80 g	Digoxin, diuretic, nitrates	4 W	①④
Gan (2019)	30	30	III-IV						59.3 ± 5.7	59.8 ± 5.6	18/12	16/14	*A. membranaceus* injection, 20 mL, qd + CT	CT	40 g	Digoxin, diuretic, nitrates, ACEI	2 W	①④⑤
Huang et al. (2016)	55	55	III-IV						66.45 ± 4.32	65.35 ± 4.65	32/23	31/24	*A. membranaceus* injection, 60 mL, qd + CT	CT	120 g	Digoxin, diuretic, nitrates	2 W	①②③⑦⑧⑨
Liu (2010)	30	22		8	22		7	15	56.2 ± 14.9	55.8 ± 13.7	17/13	13/9	*A. membranaceus* injection, 20 mL, qd + CT	CT	40 g	Digoxin, diuretic, ACEI, β-receptor blockers	2 W	①④
Lv et al. (2019)	45	45	12	33		11	34		64.3 ± 4.8	64.6 ± 5.2	25/20	28/17	*A. membranaceus* granules, 30 g, bid + CT	CT	100 g	Digoxin, diuretic, nitrates	4 W	①④⑤⑥⑦⑧⑨
Meng and Ma. (2008)	49	49		21	28		23	26	48.2	48.6	25/24	26/23	*A. membranaceus* injection, 30 mL, qd + CT	CT	60 g	ACEI, β-receptor blockers, spironolactone	12 d	①④⑥
Shen et al. (2016)	39	39	II-III						67.23 ± 6.66	68.01 ± 6.93	24/15	18/21	*A. membranaceus* injection, 20 mL, qd + CT	CT	40 g	Diuretic, ACEI, carvedilol	30 d	⑤⑦⑧
Wang et al. (2010)	78	52	24	31	23	16	21	15	62.4	62.6	44/34	28/24	*A. membranaceus* injection, 20 mL, qd + CT	CT	40 g	Digoxin, diuretic, ACEI, β-receptor blockers	2 W	①④⑤⑩
Wei (2018)	34	34	12	22		14	20		60.54 ± 8.62	60.71 ± 8.66	22/12	20/14	*A. membranaceus* granules, 30 g, bid + CT	CT	100 g	ACEI, β-receptor blockers, spironolactone	2 W	①②③④⑥
Wu and Chen. (2005)	26	26		14	12		15	11	39.10 ± 17.92	41.20 ± 16.81	13/13	15/11	*A. membranaceus* injection, 30 mL, qd + CT	CT	60 g	ACEI, β-receptor blockers, spironolactone	15 d	①④
Xian (2019)	48	48	28	20		29	19		65.27 ± 7.16	64.58 ± 7.32	26/22	27/21	*A. membranaceus* injection, 20 mL, qd + CT	CT	40 g	Aspirin, clopidogrel, ACEI	2 W	①②③④⑤⑦⑨⑩
Yang et al. (2010)	22	23	II-III						60.3 ± 4.7	62.5 ± 4.3	12/10	13/10	*A. membranaceus* granules, 30 g, bid + CT	CT	100 g	Torsemide, ACEI, β-receptor blockers, spironolactone	2 W	①⑥⑦
Yang and Tu. (2019)	51	51	11	30	10	13	27	11	63.97 ± 7.09	64.59 ± 7.28	30/21	28/23	*A. membranaceus* injection, 20 mL, qd + CT	CT	40 g	Digoxin, diuretic, nitrates	2 W	①②③④⑤
Yuan et al. (2019)	60	62	II-IV						61.4 ± 11.5	62.5 ± 11.8	29/31	26/34	*A. membranaceus* granules, 30 g, bid + CT	CT	100 g	Diuretic, ACEI, β-receptor blockers, spironolactone	4 W	①④⑤⑥
Zhang et al. (2005)	42	42	12	19	11	13	18	11	61.1 ± 9.8	60.3 ± 10.1	24/18	23/19	*A. membranaceus* injection, 30 mL, qd + CT	CT	60 g	Digoxin, diuretic, nitrates, ACEI	4 W	①⑦⑧
Zhang and Li. (2014)	35	33	10	15	10	7	13	13	63.15 ± 2.46	63.84 ± 3.28	18/17	12/11	*A. membranaceus* injection, 40 mL, qd + CT	CT	80 g	Digoxin, diuretic, ACEI, β-receptor blockers	15 d	①④⑦⑧
Zhong et al. (2016)	59	59	III-IV						66.27 ± 3.41	66.43 ± 3.52	32/27	35/24	*A. membranaceus* injection, 60 mL, qd + CT	CT	120 g	Digoxin, diuretic, nitrates	4 W	①⑤⑥
Zhu et al. (2016)	40	40	9	20	11	11	19	10	29–68	31–65	22/18	25/15	*A. membranaceus* injection, 20 mL, qd + CT	CT	40 g	Digoxin, diuretic, nitrates, ACEI, β-receptor blockers, spironolactone	4 W	①②③④⑤

Note: C, control group; T, treatment group; M, male; F, female; d, days; w, weeks; qd, quaque in die; bid, bis in die; CT: conventional treatment; ACEI: angiotensin-converting enzyme inhibitor; NYHA, New York Heart Association. Outcomes: ①LVEF; ②LVEDD; ③LVESD; ④Clinical efficacy; ⑤BNP; ⑥6-MWD; ⑦TNF-α; ⑧IL-6; ⑨hs-CRP; ⑩Adverse reactions.

### 3.3 Risk of bias assessment

**FIGURE 2:**
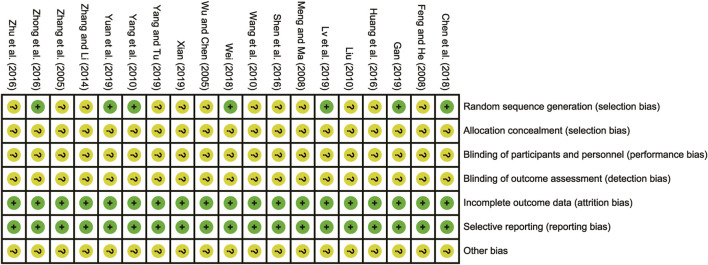
Bias risk assessment of included studies.

All the studies included were RCTs. Eight of these studies (Zhang et al., 2005; Yang et al., 2010; Zhong et al., 2016; Chen et al., 2018; Wei, 2018; Gan, 2019; Xian, 2019; Yang and Tu, 2019) reported using appropriate randomization methods, particularly the random number table method, which was designated as low risk. However, the remaining studies (Wu and Chen, 2005; Feng and He, 2008; Meng and Ma, 2008; Liu, 2010; Wang et al., 2010; Zhang and Li, 2014; Huang et al., 2016; Shen et al., 2016; Zhu et al., 2016; Lv et al., 2019; Yuan et al., 2019) did not provide clear descriptions of their randomization procedures, resulting in them being evaluated as having unclear risks. When it came to allocation concealment and blind evaluation, none of the RCTs were explicitly mentioned as having addressed these aspects, leading to their classification as having unclear risks. Nonetheless, all the included RCTs reported no bias in selective reporting regarding incomplete outcome data, thus being evaluated as low risk in this regard. The risk of bias assessment is presented in Figure 2.

### 3.4 Primary outcomes

#### 3.4.1 LVEF

**FIGURE 3:**
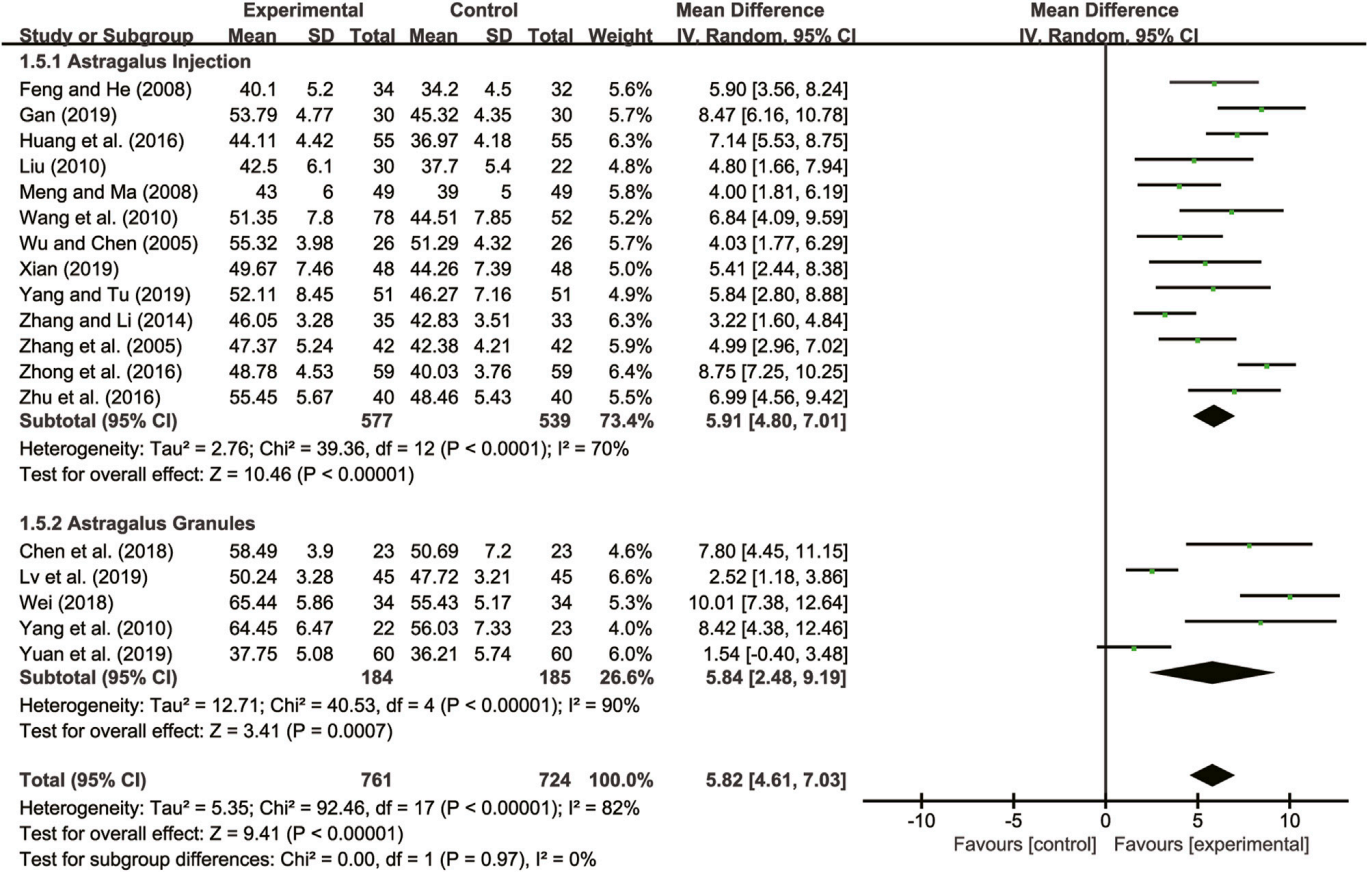
Forest plot for LVEF.

Eighteen studies (Wu and Chen, 2005; Zhang et al., 2005; Feng and He, 2008; Meng and Ma, 2008; Liu, 2010; Wang et al., 2010; Yang et al., 2010; Zhang and Li, 2014; Huang et al., 2016; Zhong et al., 2016; Zhu et al., 2016; Chen et al., 2018; Wei, 2018; Gan, 2019; Lv et al., 2019; Xian, 2019; Yang and Tu, 2019; Yuan et al., 2019) evaluated LVEF with high heterogeneity between them (*I* ^*2*^ = 82%, *p* < 0.00001) and were merged with a random-effects model. The combination therapy of *A. membranaceus* with CT significantly improved LVEF compared to CT (MD = 5.82, 95% CI: 4.61 to 7.03, *p* < 0.00001, Figure 3). A subgroup analysis based on different dosage forms of *A. membranaceus* demonstrated notable distinctions between *A. membranaceus* injection (MD = 5.91, 95% CI: 4.80 to 7.01, *p* < 0.00001, Figure 3), *A. membranaceus* granules (MD = 5.84, 95% CI: 2.48 to 9.19, *p* = 0.0007, Figure 3) and CT.

#### 3.4.2 LVEDD

**FIGURE 4:**
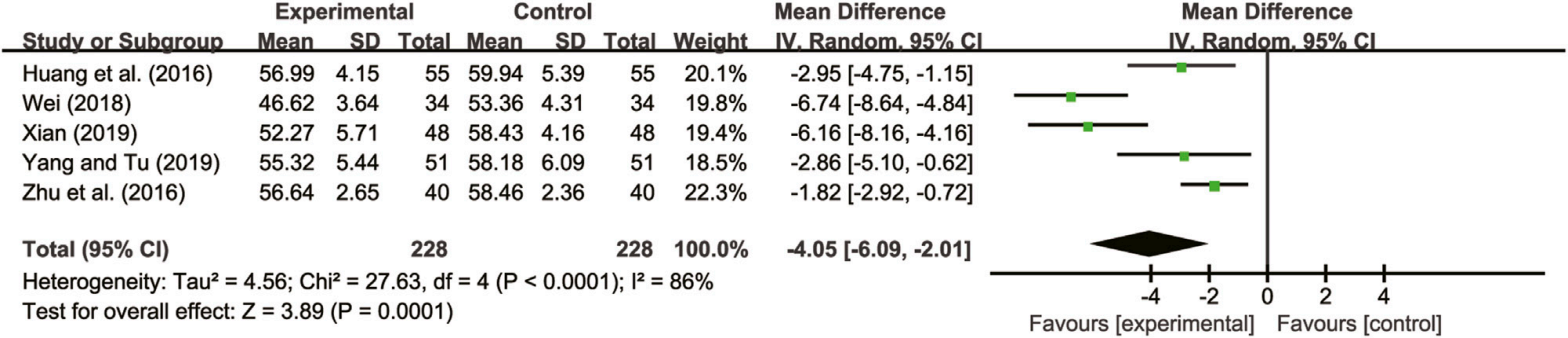
Forest plot for LVEDD.

Five studies (Huang et al., 2016; Zhu et al., 2016; Wei, 2018; Xian, 2019; Yang and Tu, 2019) evaluated LVEDD with high heterogeneity between them (*I* ^*2*^ = 86%, *p* < 0.0001) and were merged with a random-effects model. The combination therapy of *A. membranaceus* with CT significantly reduced LVEDD compared to CT (MD = −4.05, 95% CI: −6.09 to −2.01, *p* = 0.0001, Figure 4).

#### 3.4.3 LVESD

**FIGURE 5:**
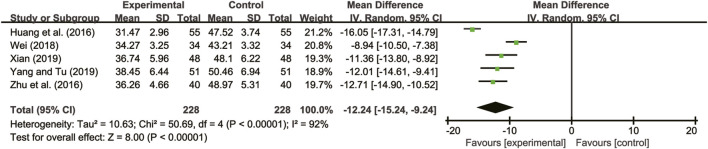
Forest plot for LVESD.

Five studies (Huang et al., 2016; Zhu et al., 2016; Wei, 2018; Xian, 2019; Yang and Tu, 2019) evaluated LVESD with high heterogeneity between them (*I* ^*2*^ = 92%, *p* < 0.00001) and were merged with a random-effects model. The combination therapy of *A. membranaceus* with CT significantly reduced LVESD compared to CT (MD = −12.24, 95% CI: −15.24 to −9.24, *p* < 0.00001, Figure 5).

### 3.5 Secondary outcomes

#### 3.5.1 Clinical efficacy

**FIGURE 6:**
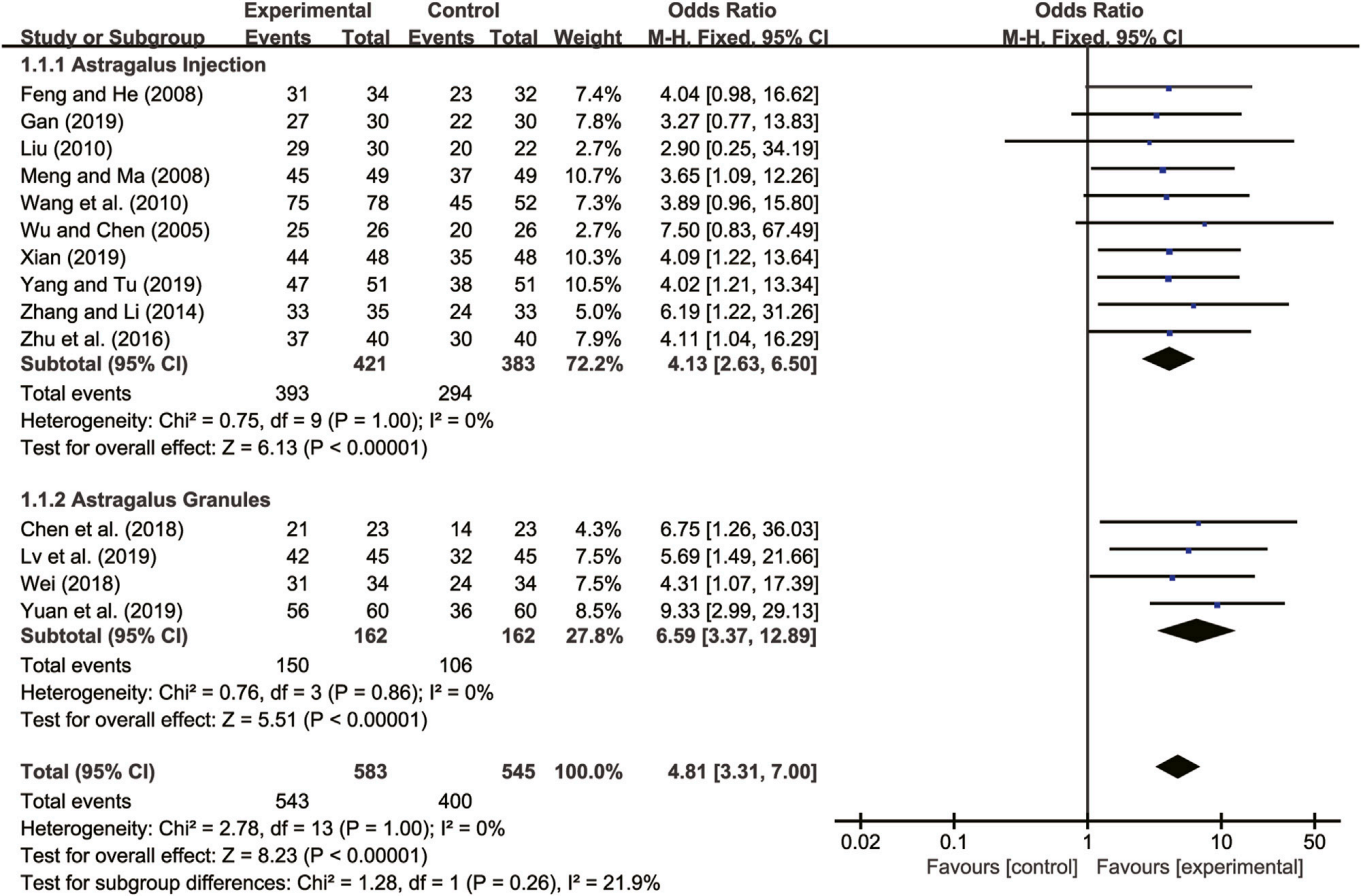
Forest plot for clinical efficacy.

Fourteen studies (Wu and Chen, 2005; Feng and He, 2008; Meng and Ma, 2008; Liu, 2010; Wang et al., 2010; Zhang and Li, 2014; Zhu et al., 2016; Chen et al., 2018; Wei, 2018; Gan, 2019; Lv et al., 2019; Xian, 2019; Yang and Tu, 2019; Yuan et al., 2019) evaluated clinical efficacy with low heterogeneity between them (*I* ^*2*^ = 0, *p =* 1.00) and were merged with a fixed-effects model. The combination therapy of *A. membranaceus* with CT significantly improved clinical efficacy compared to CT (RR = 4.81, 95% CI: 3.31 to 7.00, *p* < 0.00001, Figure 6). A subgroup analysis based on different dosage forms of *A. membranaceus* demonstrated notable distinctions between *A. membranaceus* injection (RR = 4.13, 95% CI: 2.63 to 6.50, *p* < 0.00001, Figure 6), *A. membranaceus* granules (RR = 6.59, 95% CI: 3.37 to 12.89, *p* < 0.00001, Figure 6) and CT.

#### 3.5.2 BNP

**FIGURE 7:**
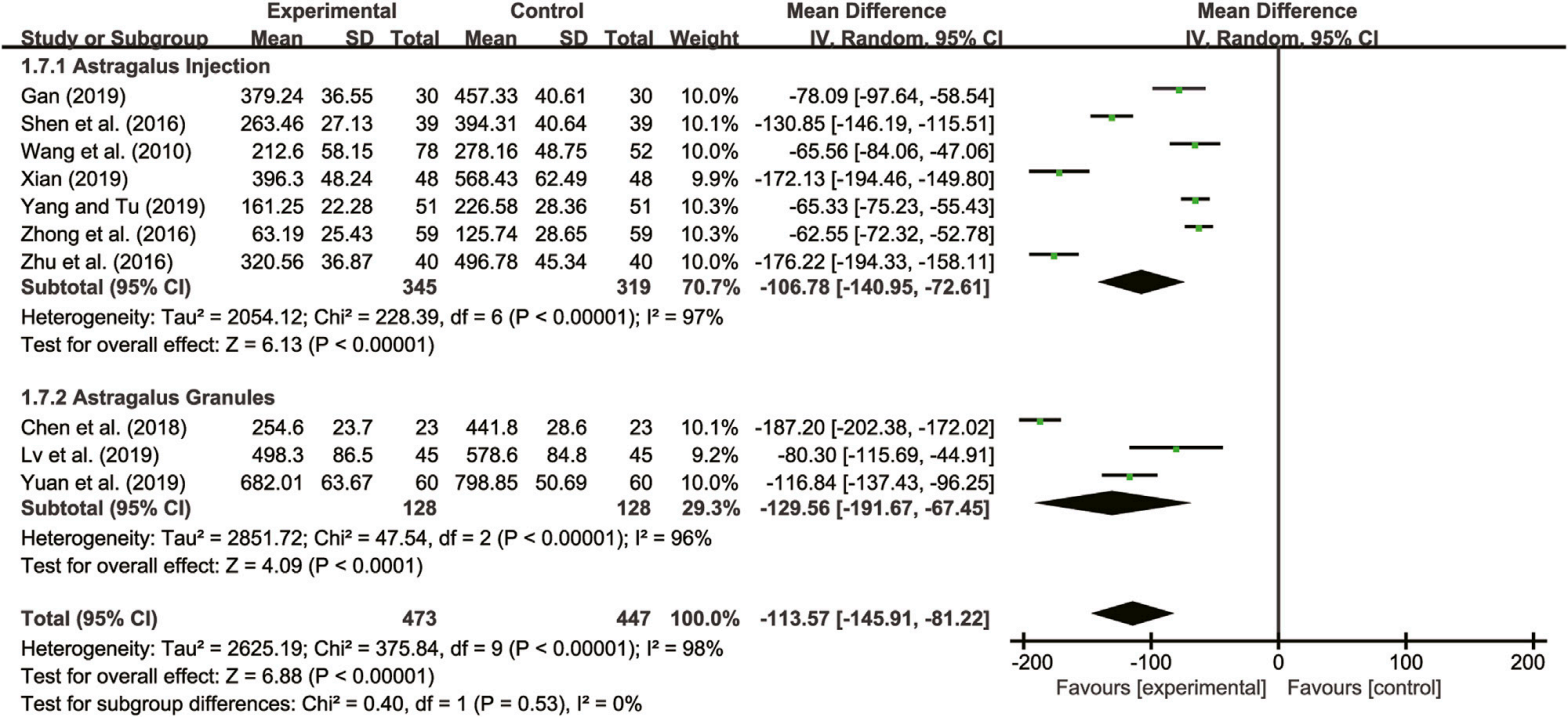
Forest plot for BNP.

Ten studies (Wang et al., 2010; Shen et al., 2016; Zhong et al., 2016; Zhu et al., 2016; Chen et al., 2018; Gan, 2019; Lv et al., 2019; Xian, 2019; Yang and Tu, 2019; Yuan et al., 2019) evaluated BNP with high heterogeneity between them (*I* ^*2*^ = 98%, *p* < 0.00001) and were merged with a random-effects model. The combination therapy of *A. membranaceus* with CT significantly reduced BNP compared to CT (MD = −113.57, 95% CI: −146.91 to −81.22, *p* < 0.00001, Figure 7). A subgroup analysis based on different dosage forms of *A. membranaceus* demonstrated notable distinctions between *A. membranaceus* injection (MD = −106.78, 95% CI: −140.95 to −72.61, *p* < 0.00001, Figure 7), *A. membranaceus* granules (MD = −129.56, 95% CI: −191.67 to −67.45, *p* < 0.00001, Figure 7) and CT.

#### 3.5.3 6-MWD

**FIGURE 8:**
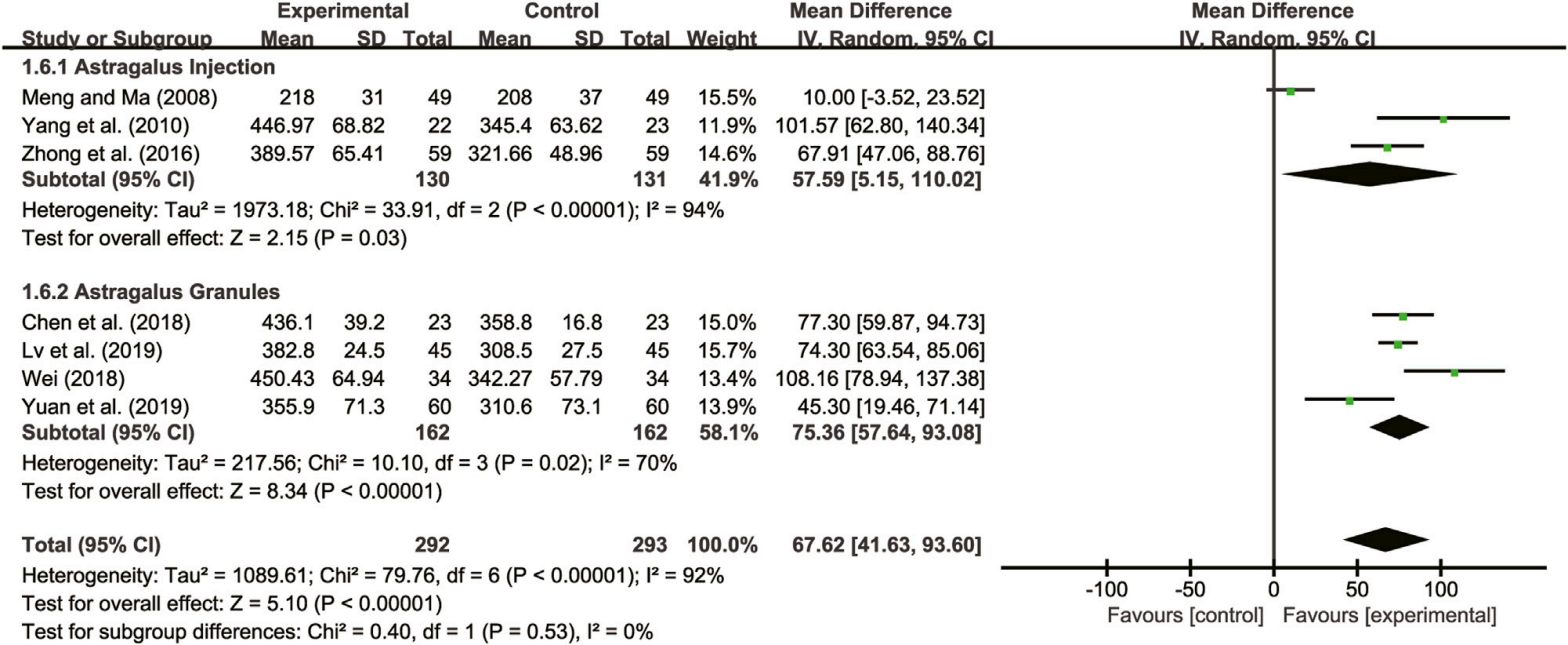
Forest plot for 6-MWD.

Seven studies (Meng and Ma, 2008; Yang et al., 2010; Zhong et al., 2016; Chen et al., 2018; Wei, 2018; Lv et al., 2019; Yuan et al., 2019) evaluated 6-MWD with high heterogeneity between them (*I* ^*2*^ = 92%, *p* < 0.00001) and were merged with a random-effects model. The combination therapy of *A. membranaceus* with CT significantly reduced 6-MWD compared to CT (MD = 67.62, 95% CI: 41.63 to 93.60, *p* < 0.00001, Figure 8). A subgroup analysis based on different dosage forms of *A. membranaceus* demonstrated notable distinctions between *A. membranaceus* injection (MD = 57.59, 95% CI: 5.15 to 110.02, *p* < 0.00001, Figure 8), *A. membranaceus* granules (MD = 75.36, 95% CI: 57.64 to 93.08, *p* < 0.00001, Figure 8) and CT.

#### 3.5.4 TNF-α

**FIGURE 9:**
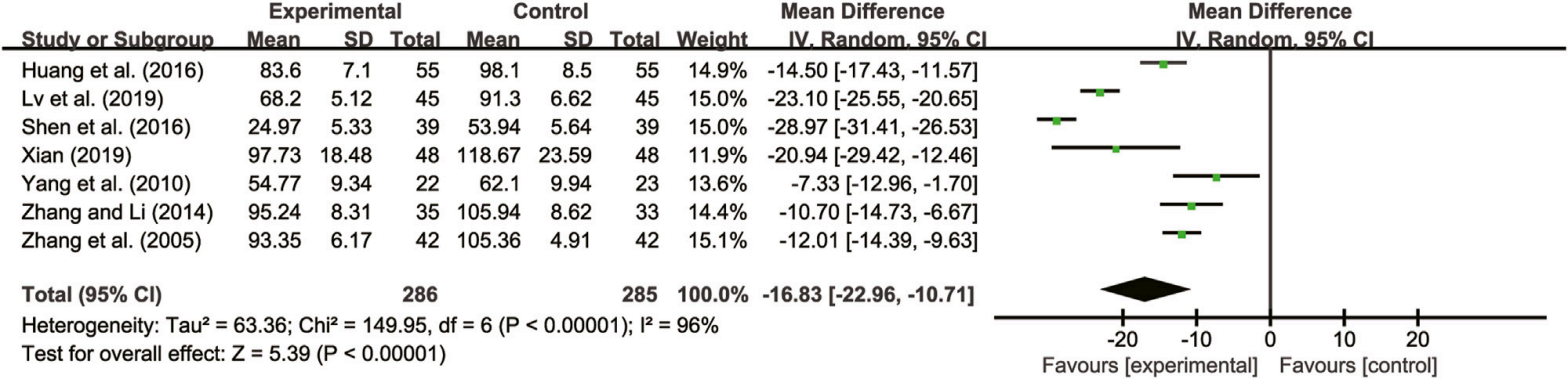
Forest plot for TNF-α.

Seven studies (Zhang et al., 2005; Yang et al., 2010; Zhang and Li, 2014; Huang et al., 2016; Shen et al., 2016; Lv et al., 2019; Xian, 2019) evaluated TNF-α with high heterogeneity between them (*I* ^*2*^ = 96%, *p* < 0.00001) and were merged with a random-effects model. The combination therapy of *A. membranaceus* with CT significantly reduced TNF-α compared to CT (MD = −16.83, 95% CI: −22.96 to −10.71, *p* < 0.00001, Figure 9).

#### 3.5.5 IL-6

**FIGURE 10:**
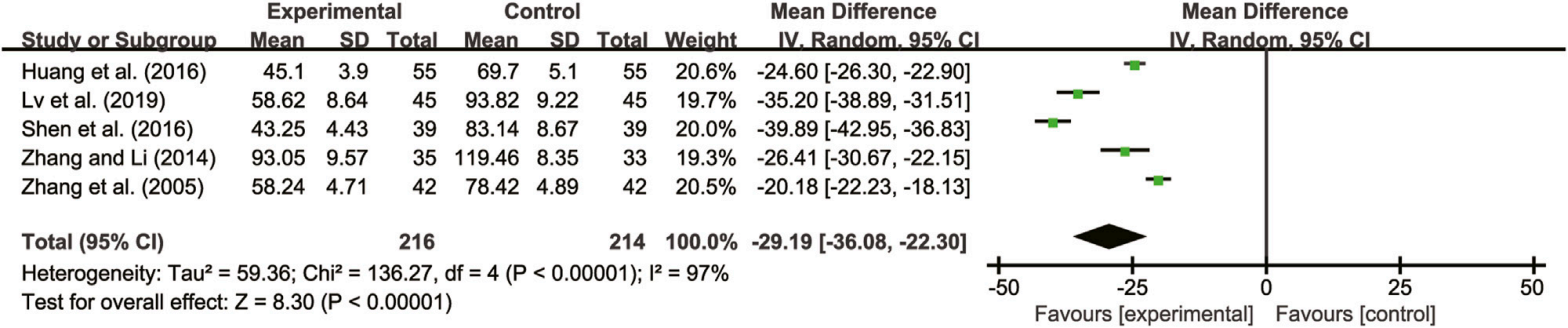
Forest plot for IL-6.

Five studies (Zhang et al., 2005; Zhang and Li, 2014; Huang et al., 2016; Shen et al., 2016; Lv et al., 2019) evaluated IL-6 with high heterogeneity between them (*I* ^*2*^ = 97%, *p* < 0.00001) and were merged with a random-effects model. The combination therapy of *A. membranaceus* with CT significantly reduced IL-6 compared to CT (MD = −29.19, 95% CI: −36.08 to −22.30, *p* < 0.00001, Figure 10).

#### 3.5.6 hs-CRP

**FIGURE 11:**
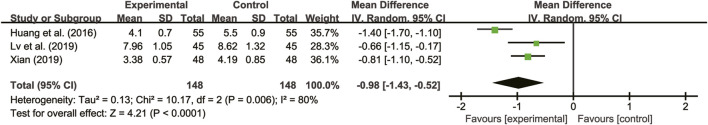
Forest plot for hs-CRP.

Three studies (Huang et al., 2016; Lv et al., 2019; Xian, 2019) evaluated hs-CRP with high heterogeneity between them (*I* ^*2*^ = 80%, *p* = 0.06) and were merged with a random-effects model. The combination therapy of *A. membranaceus* with CT significantly reduced hs-CRP compared to CT (MD = −0.98, 95% CI: −1.43 to −0.52, *p* < 0.00001, Figure 11).

#### 3.5.7 Adverse reactions

**FIGURE 12:**
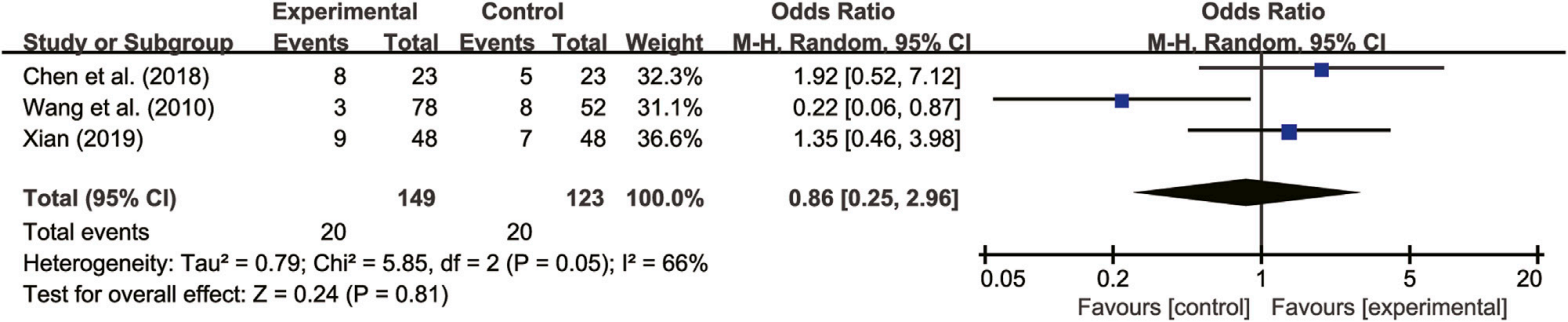
Forest plot for adverse reactions.

Seven studies (Wu and Chen, 2005; Meng and Ma, 2008; Wang et al., 2010; Yang et al., 2010; Chen et al., 2018; Wei, 2018; Xian, 2019) reported on adverse reactions. Four of these studies (Wang et al., 2010; Chen et al., 2018; Xian, 2019) did not detect any adverse reactions, while the remaining three studies (Wang et al., 2010; Chen et al., 2018; Xian, 2019) showed high heterogeneity (*I* ^*2*^ = 66%, *p* = 0.05). A random-effects model analysis revealed no significant difference in adverse reactions between patients treated with *A. membranaceus* combination with CT and those receiving CT (RR = 0.86, 95% CI = 0.25 to 2.96, *p* = 0.81, Figure 12). Common adverse reactions included hypotension, sinus bradycardia, nausea, dizziness, and cough, which usually disappeared with symptom management. Notably, no participant discontinued the study drug due to adverse reactions. The details are presented in Table 2.

**TABLE 2:** The incidence rate of adverse reactions.

Adverse reaction symptoms	First author (publication year)	The number of adverse reactions	
		T	C
Hypotension	Chen et al. (2018); Wang et al. (2010); Xian (2019)	9	7
Sinus Bradycardia	Chen et al. (2018); Wang et al. (2010); Xian (2019)	6	6
Nausea	Xian (2019)	2	3
Dizziness	Wang et al. (2010)	3	0
Cough	Wang et al. (2010)	0	4
Total reactions	-	20/149	20/123
Incidence rate	-	13.42%	16.26%

### 3.6 Sensitivity analysis

**FIGURE 13:**
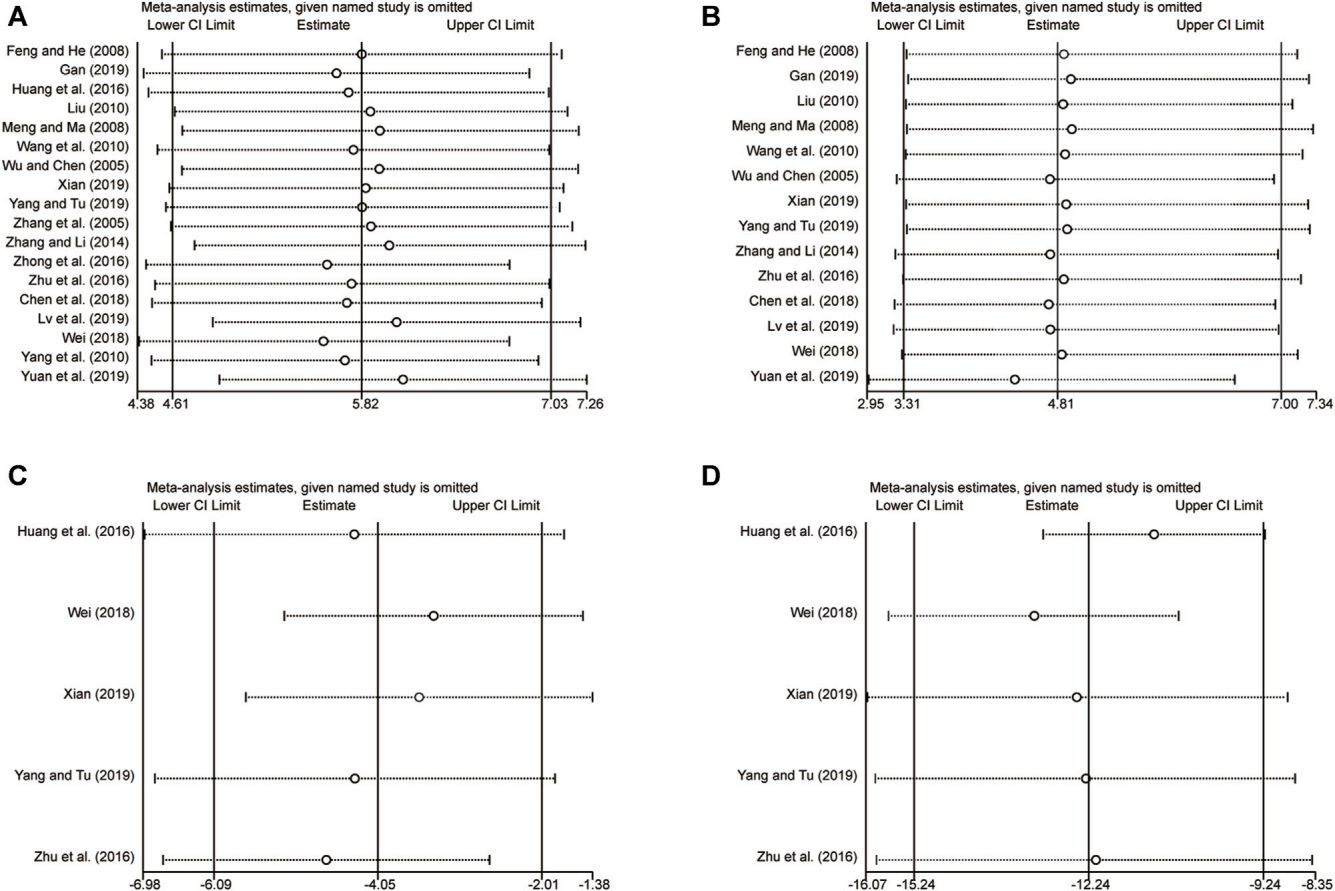
The results of sensitivity analysis. **(A)** LVEF. **(B)** Clinical efficacy. **(C)** LVEDD. **(D)** LVESD.

Sensitivity analysis was performed by sequentially removing individual studies to assess their impact on the overall merged results. The analysis revealed that excluding any individual studies for LVEF (Figure 13A), clinical efficacy (Figure 13B), LVEDD (Figure 13C), and LVESD (Figure 13D) had no effect on the merged results. This suggests that the merged results are robust and reliable, as indicated in Figure 13.

### 3.7 Publication bias

**FIGURE 14:**
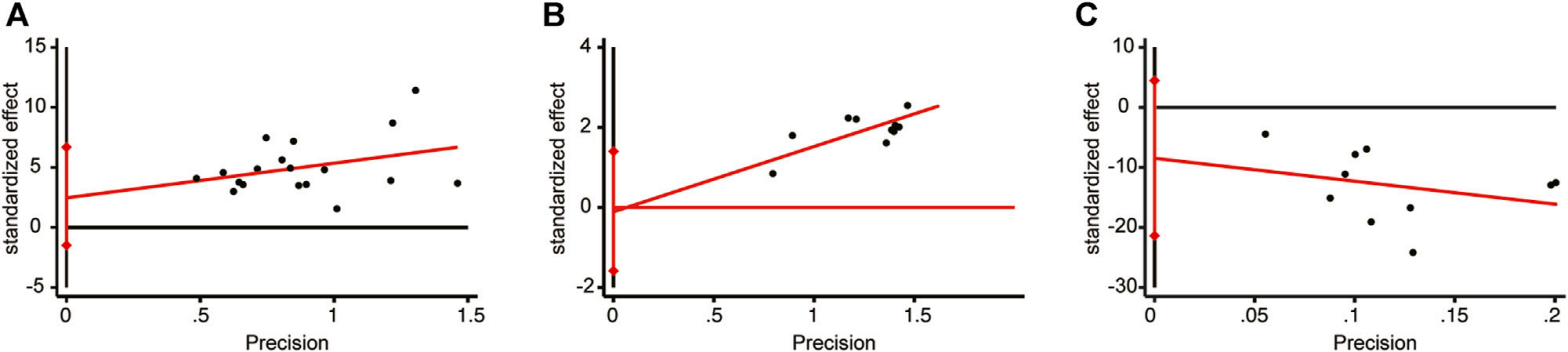
Egger’s publication funnel plot. **(A)** LVEF. **(B)** Clinical efficacy. **(C)** BNP.

The meta-analysis for LVEF, clinical efficacy, and BNP included a minimum of 10 studies. These three indicators were chosen to assess publication bias using the Egger’s test (Figure 14). The results revealed no significant publication bias for LVEF (*p* = 0.211), clinical efficacy (*p* = 0.877), or BNP (*p* = 0.168).

## 4 Discussion

### 4.1 Summary of findings

This meta-analysis analyzed 19 RCTs to investigate the effect of *A. membranaceus* on LVR in patients with HFrEF. The primary findings can be distilled into four key observations: (1) The combination of *A. membranaceus* and CT significantly improved LVR. This was exemplified by an increase in LVEF, a decrease in LVEDD and LVESD, and lower BNP level. (2) The combination therapy of *A. membranaceus* with CT significantly enhanced the clinical efficacy, resulting in an increase in 6-MWD. (3) The combination therapy of *A. membranaceus* with CT notably reduced the inflammatory response, demonstrated by decreased expression levels of TNF-α, IL-6, and hs-CRP. (4) The combination therapy of *A. membranaceus* with CT demonstrated a good safety, with only mild adverse events reported. These events were manageable symptomatically and had no impact on treatment outcomes.

The sensitivity analysis, conducted by sequentially deleting individual studies, confirmed that the merged results remained robust and reliable. However, the source of heterogeneity could not be pinpointed, which was possibly due to factors such as the limited inclusion of studies and sample size. To assess publication bias, an analysis using STATA 17.0 software revealed that there was no significant bias in the published studies.

### 4.2 Comparison with previous studies

Up to now, systematic reviews and meta-analyses published by Bo et al. and Li et al. (Bo et al., 2019; Li et al., 2019) have demonstrated that the combination of traditional Chinese medicine (TCM) containing *A. membranaceus* and CT can further improve clinical efficacy in treating HF compared to CT. However, there are some limitations to the published research.

Firstly, HF is classified into HFrEF and heart failure with preserved ejection fraction (HFpEF) based on ejection fraction. However, previous studies included in the meta-analysis did not classify HF based on ejection fraction, and subgroup analysis was not conducted during the meta-analysis, which may increase the risk of bias. Secondly, ventricular remodeling is a key mechanism in the onset and progression of HF (Pezel et al., 2021). Previous meta-analyses focused on evaluating the clinical efficacy and safety of TCM preparations containing *A. membranaceus* on HF, but did not analyze whether *A. membranaceus* has advantages in improving ventricular remodeling in HF. Once again, existing evidence have confirmed that chronic inflammatory response is an important factor in the progression of HF (Beydoun and Feinstein, 2022), which has not been emphasized in previous published studies.

### 4.3 Strengths and limitations

This study offers several strengths: (1) By focusing on HFrEF patients, this study eliminates the potential impact of disease type on results, distinguishing it from previous studies on the treatment of HF with *A. membranaceus*. (2) This study goes beyond previous research by evaluating the effect of *A. membranaceus* on LVR in HFrEF patients. It also conducts sensitivity analysis on key indicators of LVR, such as LVEF, LVEDD, and LVESD, enhancing the reliability of results. (3) Inflammation, an important pathological factor in LVR, was evaluated in this study. This component was previously unaddressed in previous studies.

However, our study is not without its limitations: 1) Some studies only mentioned the severity of HF without providing specific numbers, which could lead to potential heterogeneity. 2) The included studies did not describe random concealment and blinding, which could have resulted in selective bias. 3) Although detailed searches were conducted in both Chinese and English databases, all RCTs included were conducted in China, which could be a source of bias. 4) Only 7 of the 19 studies reported adverse reactions, so the safety of *A. membranaceus* intervention requires further investigation. 5) The inclusion of studies without reporting follow-up time may have led to an insufficient evaluation of the results. 6) Some of the RCTs included in this study are relatively small in scale, and larger-scale research is still needed to ensure the reliability of the results. 7) Given the moderate to high heterogeneity of our results, sensitivity analysis alone is not sufficient to draw definitive conclusions. Therefore, further research is imperative to validate and establish the reliability of our findings.

### 4.4 Implication

The implications for future research are as follows: Firstly, HF should be classified according to guidelines, including HFrEF and HFpEF. More high-quality RCTs should be conducted to ensure more accurate conclusions. Secondly, strongly encourage the conduct of placebo RCTs to facilitate blinding and reduce publication bias. Thirdly, the complete report of the research results should be based on the comprehensive standard report trial statement. In addition to outcome indicators, the focus should also be on reporting the course of disease, NYHA classification, comorbidities, readmission rate, and follow-up time to identify the source of heterogeneity. Fourthly, long-term follow-up is necessary, especially for endpoint events and safety outcomes. Fifthly, sodium glucose co-transporter 2 (SGLT2) inhibitors have emerged as a promising approach to improve LVR (Carluccio et al., 2023). Nevertheless, there is currently a substantial lack of research investigating the combination therapy involving *A. membranaceus* and SGLT2 inhibitors for HF. Future research efforts should focus on exploring the potential benefits and synergistic effects of this combination therapy.

## 5 Conclusion

Existing evidence indicates that the combination of *A. membranaceus* with CT has advantages in improving LVR in HFrEF patients compared to CT alone, and it is generally safer. However, given the existence of some risk factors, including the course of HF, allocation concealment, blinding, and follow-up time, future RCTs should report their results based on the CONSORT statement to minimize the risk of bias and provide more substantial evidence.

## Data Availability

The original contributions presented in the study are included in the article/Supplementary Material, further inquiries can be directed to the corresponding author.

## References

[B1] Aimo A. Gaggin H. K. Barison A. Emdin M. Januzzi J. L. Jr. (2019). Imaging, biomarker, and clinical predictors of cardiac remodeling in heart failure with reduced ejection fraction. JACC Heart Fail 7 (9), 782–794. 10.1016/j.jchf.2019.06.004 31401101

[B2] Álvarez-Zaballos S. Martínez-Sellés M. (2023). Angiotensin-converting enzyme and heart failure. Front. Biosci. (Landmark Ed. 28 (7), 150. 10.31083/j.fbl2807150 37525924

[B3] Beydoun N. Feinstein M. J. (2022). Heart failure in chronic infectious and inflammatory conditions: mechanistic insights from clinical heterogeneity. Curr. Heart Fail Rep. 19 (5), 267–278. 10.1007/s11897-022-00560-3 35838874 PMC9283814

[B4] Biering-Sørensen T. Minamisawa M. Claggett B. Liu J. Felker G. M. McMurray J. J. V. (2020). Cardiac myosin activator omecamtiv mecarbil improves left ventricular myocardial deformation in chronic heart failure: the COSMIC-HF trial. Circ. Heart Fail 13 (12), e008007. 10.1161/circheartfailure.120.008007 33176443

[B5] Bloom M. W. Greenberg B. Jaarsma T. Januzzi J. L. Lam C. S. P. Maggioni A. P. (2017). Heart failure with reduced ejection fraction. Nat. Rev. Dis. Prim. 3, 17058. 10.1038/nrdp.2017.58 28836616

[B6] Bo Y. Zhang P. Li Z. Lu J. Lu B. (2019). Meta-analysis of the therapeutic effect of *Astragalus membranaceus* medicines on chronic heart failure. J. Emerg. Tradit. Chin. Med. 28 (04), 637–640.

[B7] Carluccio E. Biagioli P. Reboldi G. Mengoni A. Lauciello R. Zuchi C. (2023). Left ventricular remodeling response to SGLT2 inhibitors in heart failure: an updated meta-analysis of randomized controlled studies. Cardiovasc Diabetol. 22 (1), 235. 10.1186/s12933-023-01970-w 37660005 PMC10475184

[B8] Chen P. Y. Wang Y. B. Wang B. D. (2018). Clinical efficacy of *Astragalus membranaceus* granules combined with Benazepril in the treatment of dilated cardiomyopathy. Chin. J. Mod. Drug Appl. 12 (09), 1–3. 10.14164/j.cnki.cn11-5581/r.2018.09.001

[B9] Cumpston M. Li T. Page M. J. Chandler J. Welch V. A. Higgins J. P. (2019). Updated guidance for trusted systematic reviews: a new edition of the Cochrane Handbook for Systematic Reviews of Interventions. Cochrane Database Syst. Rev. 10 (10), Ed000142. 10.1002/14651858.Ed000142 31643080 PMC10284251

[B10] Dai H. Tao S. Guan Y. Zhang Y. Yang Z. Jia J. (2023). Astragalus (Astragalus mongholicus) improves ventricular remodeling via ESR1 downregulation RhoA/ROCK pathway. Int. Heart J. 64 (6), 1148–1156. 10.1536/ihj.23-265 37967985

[B11] Feng L. Y. He W. (2008). Clinical effects of *Astragalus membranaceus* injection in treatment of 34 patients with chronic heart failure. Chin. J. Integr. Med. Cardio/ Cerebrovasc. Dis. (11), 1362–1363.

[B12] Gan P. Z. (2019). Effects of *Astragalus membranaceus* injection in the treatment of chronic heart failure and its effect on BNP and CA125. Chin. Med. Pharm. 9 (09), 48–51.

[B13] Huang J. Yang J. Luo Y. H. Qu W. C. (2016). Effects of *Astragalus membranaceus* on cardiac function, cell cytokines, and inflammatory factors in the treatment of chronic heart failure. Chin. J. Mod. Drug Appl. 10 (24), 94–96. 10.14164/j.cnki.cn11-5581/r.2016.24.059

[B14] Hutton B. Salanti G. Caldwell D. M. Chaimani A. Schmid C. H. Cameron C. (2015). The PRISMA extension statement for reporting of systematic reviews incorporating network meta-analyses of health care interventions: checklist and explanations. Ann. Intern Med. 162 (11), 777–784. 10.7326/m14-2385 26030634

[B15] Lavalle C. Di Lullo L. Jabbour J. P. Palombi M. Trivigno S. Mariani M. V. (2023). New challenges in heart failure with reduced ejection fraction: managing worsening events. J. Clin. Med. 12 (22), 6956. 10.3390/jcm12226956 38002571 PMC10672118

[B16] Lee M. M. Y. Brooksbank K. J. M. Wetherall K. Mangion K. Roditi G. Campbell R. T. (2021). Effect of empagliflozin on left ventricular volumes in patients with type 2 diabetes, or prediabetes, and heart failure with reduced ejection fraction (SUGAR-DM-HF). Circulation 143 (6), 516–525. 10.1161/circulationaha.120.052186 33186500 PMC7864599

[B17] Li W. Li S. Han J. Tan Y. Wang L. Xian S. (2019). The hemodynamic effects of *Astragalus membranaceus* injection in the treatment of chronic heart failure: a meta-analysis of clinical controlled trials. Chin. Med. J. Res. Prac. 33 (01), 63–68. 10.13728/j.1673-6427.2019.01.015

[B18] Liu T. H. (2010). Clinical observation of the effect of Sodium Nitroprusside combining with *Astragalus membranaceus* injection on chronic heart failure. Natl. Med. Front. Chin. 5 (04), 20–21.

[B19] Lv Z. Y. Zhu X. H. Li H. J. (2019). Clinical study on *Astragalus membranaceus* granules in treatment of vascular inflammation in patients with chronic heart failure. Chin. J. Evid. Based Cardiovasc Med. 11 (09), 1089–1091.

[B20] Ma J. Qiao Z. Xiang X. (2011). Aqueous extract of Astragalus mongholicus. J. Med. Plants Res. 5 (5), 855–858.

[B21] Meng H. H. Ma M. J. (2008). Clinical observation of the effect of *Astragalus membranaceus* injection on chronic heart failure. Clin. J. Tradit. Chin. Med. 20 (01), 40–41. 10.16448/j.cjtcm.2008.01.037

[B22] Pezel T. Viallon M. Croisille P. Sebbag L. Bochaton T. Garot J. (2021). Imaging interstitial fibrosis, left ventricular remodeling, and function in stage A and B heart failure. JACC Cardiovasc Imaging 14 (5), 1038–1052. 10.1016/j.jcmg.2020.05.036 32828781

[B23] Ren C. Zhao X. Liu K. Wang L. Chen Q. Jiang H. (2023). Research progress of natural medicine Astragalus mongholicus Bunge in treatment of myocardial fibrosis. J. Ethnopharmacol. 305, 116128. 10.1016/j.jep.2022.116128 36623754

[B24] Roger V. L. (2021). Epidemiology of heart failure: a contemporary perspective. Circ. Res. 128 (10), 1421–1434. 10.1161/circresaha.121.318172 33983838

[B25] Shen S. Q. Zhao M. Q. Zhang K. K. (2016). Effect of *Astragalus membranaceus* injection combined with β-adrenoceptor antagonist on BNP, IL-1β, IL-6 and TNF-α levels in heart failure patients. Chin. J. Biochem. Pharm. 36 (04), 150–151+154.

[B26] Stencel J. Alai H. R. Dhore-Patil A. Urina-Jassir D. Le Jemtel T. H. (2023). Obesity, preserved ejection fraction heart failure, and left ventricular remodeling. J. Clin. Med. 12 (9), 3341. 10.3390/jcm12093341 37176781 PMC10179420

[B27] Wang H. H. Zhu Y. P. Deng Y. Zhang F. Y. Han L. (2010). Clinical study on the treatment of congestive heart failure with *Astragalus membranaceus* injection. J. New Chin. Med. 42 (01), 13–15. 10.13457/j.cnki.jncm.2010.01.049

[B28] Wang P. Wang Z. Zhang Z. Cao H. Kong L. Ma W. (2023). A review of the botany, phytochemistry, traditional uses, pharmacology, toxicology, and quality control of the Astragalus memeranaceus. Front. Pharmacol. 14, 1242318. 10.3389/fphar.2023.1242318 37680711 PMC10482111

[B29] Wei D. X. (2018). Analysis of the application effect of *Astragalus membranaceus* granules in the treatment of chronic heart failure. Jiangxi J. Trad. Chin. Med. 49 (09), 47–49.

[B30] Wu X. X. Chen K. W. (2005). Effects of *Astragalus membranaceus* injectionin treatment of 26 patients with dilated cardiomyopathy. Yunnan J. Tradit. Chin. Med. Mater Medica 26 (01), 13. 10.16254/j.cnki.53-1120/r.2005.01.013

[B31] Xian W. (2019). Influence of recombinant human BNP combined Astragalus injection on heart function and serum levels of inflammatory factors in AMI patients with post-PCI heart failure. Chin. J. Cardiovasc Rehabil. Med. 28 (04), 424–428.

[B32] Xie Y. Wei Y. Li D. Pu J. Ding H. Zhang X. (2023). Mechanisms of SGLT2 inhibitors in heart failure and their clinical value. J. Cardiovasc Pharmacol. 81 (1), 4–14. 10.1097/fjc.0000000000001380 36607775

[B33] Yang H. Y. Tu Z. Q. (2019). Effects of *Astragalus membranaceus* injection combined with Torasemide on patients with renal insufficiency and heart failure, and its effects on Scr, BNP, and VEGF. Mod. J. Integr. Tradit. Chin. West Med. 28 (11), 1195–1198.

[B34] Yang J. Wang H. X. Zhang Y. J. Yang Y. H. Lu M. L. Zhang J. (2013). Astragaloside IV attenuates inflammatory cytokines by inhibiting TLR4/NF-кB signaling pathway in isoproterenol-induced myocardial hypertrophy. J. Ethnopharmacol. 150 (3), 1062–1070. 10.1016/j.jep.2013.10.017 24432369

[B35] Yang Q. Y. Lu S. Sun H. R. (2010). Effects of *Astragalus membranaceus* on cardlac function and serum TNF- α level in padents with chronic heart failure. Chin. J. Integr. Med. 30 (07), 699–701. 20929124

[B36] Yuan J. Q. Zhou A. M. Zhu Y. W. Qiu K. K. Wu X. J. (2019). Short term efficacy observation of *Astragalus membranaceus* granules in the treatment of chronic heart failure. Zhejiang J. Chin. Med. 54 (10), 723. 10.13633/j.cnki.zjtcm.2019.10.012

[B37] Zhang H. Li Z. (2014). Effect of *Astragalus membranaceus* injection on serum TNF-α, IL-6 and Ang-II in patients with chronic heart failure. J. Clin. Med. Pract. 18 (23), 17–19.

[B38] Zhang J. G. He H. Meng H. H. Wei G. H. Yang N. Wang X. Z. (2005). Effects of *Astragalus membranaceus* injection on TNF-α and IL-6 in patients with congestive heart failure. China Arch. Trad. Chin. Med. (11), 170–172. 10.13193/j.archtcm.2005.11.170.zhangjg.092

[B39] Zhong C. H. luo X. B. Li L. (2016). Observation of clinical effects of *Astragalus membranaceus* injection in treatment of 59 patients with chronic heart failure. Med. J. Chin. Pla 28 (12), 60–62.

[B40] Zhu H. He B. Yuan P. (2016). Clinical effects of *Astragalus membranaceus* in treatment of 40 patients with dilated cardiomyopathy. Henan Trad. Chin. Med. 36 (08), 1349–1351. 10.16367/j.issn.1003-5028.2016.08.0552

[B41] Zhu Y. Chai Y. Xiao G. Liu Y. Xie X. Xiao W. (2022). Astragalus and its formulas as a therapeutic option for fibrotic diseases: pharmacology and mechanisms. Front. Pharmacol. 13, 1040350. 10.3389/fphar.2022.1040350 36408254 PMC9669388

